# A Low-Cost Device for Measuring Non-Nutritive Sucking in Newborns

**DOI:** 10.3390/s25165080

**Published:** 2025-08-15

**Authors:** Sebastian Lobos, Eyleen Spencer, Pablo Reyes, Alejandro Weinstein, Jana Stojanova, Felipe Retamal-Walter

**Affiliations:** 1 PhD Program in Health Sciences and Engineering, Universidad de Valparaíso, Valparaíso 2540064, Chile; sebastian.lobos@uv.cl; 2 School of Biomedical Engineering, Faculty of Engineering, Universidad de Valparaíso, Valparaíso 2362905, Chile; eyleen.spencer@uv.cl (E.S.); pablo.reyes@uv.cl (P.R.); 3 Department of Electronic Engineering, Universidad Técnica Federico Santa Maria, Valparaíso 2390123, Chile; alejandro.weinstein@usm.cl; 4 Department of Clinical Pharmacology and Toxicology, St. Vincent’s Hospital, Sydney, NSW 2010, Australia; jana.stojanova@svha.org.au; 5 School of Health and Rehabilitation Sciences, The University of Queensland, Brisbane, QLD 4072, Australia

**Keywords:** force sensing resistors, non-nutritive sucking, piezoresistive pressure sensors

## Abstract

Non-nutritive sucking (NNS) is an instinctive behavior in newborns, consisting of two stages: sucking and expression. It plays a critical role in preparing the infant for oral feeding. In neonatal and pediatric units, NNS assessment is routinely performed to determine feeding readiness. However, these evaluations are often subjective and rely heavily on the clinician’s experience. While other medical devices that support the development of NNS skills exist, they are not specifically designed for the comprehensive assessment of NNS, and their high cost limits accessibility for many hospitals and tertiary care units globally. This paper presents the development and pilot testing of a low-cost, portable device and accompanying software for assessing NNS in newborns hospitalized in neonatal care units. Methods: The device uses force-sensitive resistors to capture expression pressure and a differential pressure sensor to measure NNS. Data were acquired through the analog–digital converter of a microcontroller and transmitted via Bluetooth for real-time graphical analysis. Pilot testing was conducted with six hospitalized preterm newborns, measuring intensity, number of bursts, and sucks per burst. Results demonstrated that the system reliably captures both stages of NNS. Significance: This device provides an affordable, portable solution to support clinical decision-making in clinical units, facilitating accurate, objective monitoring of feeding readiness and developmental progression.

## 1. Introduction

Objective and continuous assessment of newborn well-being represents a fundamental pillar of modern neonatal care, directly influencing clinical decision-making and early intervention strategies. The development of portable, non-invasive devices for monitoring multiple physiological parameters has gained momentum in recent years [1,2]. Advanced wearable and flexible biosensing systems, including on-skin optoelectronic biosensors and multifunctional e-skin technologies, are establishing new standards for next-generation healthcare solutions through real-time, non-invasive, multi-parametric physiological assessment capabilities. These technological advances hold particular relevance for neonatal and pediatric applications, where minimizing invasiveness while maximizing patient comfort remains critical. The present work aligns directly with these global healthcare trends by focusing on the design and validation of a portable system that enables simultaneous and independent measurement of key physiological parameters in newborns.

The assessment and measurement of a newborn’s non-nutritive sucking (NNS) help to determine when a newborn is capable of independently acquiring breast milk; this is especially relevant for promoting preterm infants’ growth and development, including their appropriate nutrition [3]. In the absence of technology capable of performing the same assessment, the most common method for evaluating an infant’s NNS involves inserting an examiner’s finger into the infant’s mouth. The examiner then determines the intensity of the pressure applied by the newborn on the finger when the sucking mechanism is triggered by the examiner. The subjective nature of this measurement results in great inter- and even intra-rater variability [4]. The NNS is a complex muscular activity generated by the coordinated action of the muscles of the lips, tongue, palate, and jaw. It consists of two alternating stages: sucking and expression. During the sucking stage, negative pressure is produced by the backward movement of the tongue, which increases the volume of the oral cavity, as well as by the sealing of the lips around the teat. During the expression stage, positive pressure is produced by the contraction of the peri-orbicular muscle of the lips [5]. NSS develops when the newborn is still in the womb and is fully developed prior to birth, preparing them to be fed orally through breastfeeding or bottle feeding. Adequate development of anatomical structures of the stomatognathic system involved in the feeding process is required for this phenomenon to successfully occur [6]. When newborns are born prematurely, they may have underdeveloped or ineffective NNS skills, resulting in the need for alternative means of feeding, such as tube-feeding or parenteral feeding [7]. As the development of NNS skills is impacted by anatomical and physiological factors, health professionals assess newborns’ NNS skills to determine their characteristics, stage of development, and the infant’s readiness for oral feeding.

Several authors have proposed methods to objectively measure both the sucking and expression stages of an infant’s NSS skills. For example, Rendon-Macias et al. [8] measured sucking volume, pressure, and frequency in healthy newborns. In this study, measures were gathered using an instrument consisting of a set of air ducts connected to a latex pacifier and an inspiratory pressure sensor from mechanical ventilation devices. Similarly, Lau and Kusnierczyk [4] used catheters directly connected to an examiner’s hand in order to measure both sucking and expression stages. Another study by Grassi et al. [9] integrated two digital barometric sensors inside a pacifier to measure expression and sucking, with data being received by a purpose-built device. Nishi et al. [10] developed a pacifier with built-in force sensors to measure the contact force exerted by the tongue, which only measured the expression stage of NNS. A similar device was used by Zimmerman [11,12,13] to evaluate NNS under different intervention contexts. Tam et al. [14] developed a wireless device to measure NNS in hospitalized newborns, and the device developed by Westemeyer et al. [15] has limited clinical application due to the need for a bulky data acquisition center (DAC) and a modular sensor housing. Furthermore, the system captures only the expression phase of NNS, relying solely on positive pressure changes within a pacifier without addressing the suction stage.

Although the authors and devices described earlier are capable of measuring the NNS stages, most have been developed using laboratory equipment and designed for research environments rather than clinical settings. Therefore, there are several gaps in the clinical applicability of some of these devices as they have not yet been used within neonatal or pediatric care units and/or piloted by specialized healthcare professionals who comprehensively consider the medical condition and characteristics of the newborns being assessed. Furthermore, infection prevention and control and design factors, such as the electromedical safety [16], compatibility with sterilization procedures and infection control [14,17,18], or usability characteristics [16,19], have not been fully described or incorporated in the aforementioned works. To the best of our knowledge, only two devices have been purposefully designed to examine NNS in clinical settings: the NTrainer System (Innara Health Inc., Olathe, KS, USA) [20] and the Pacifier Activated Lullaby (P.A.L.; Neolight, Scottsdale, AR, USA) [21]. The first device, the NTrainer, is primarily used to measure the outcomes of oral stimulation therapy and only captures the expression stage, thereby limiting the assessment of the complete NNS process [22]. According to its developers, the second device, the P.A.L., reinforces effective non-nutritive sucking in premature infants through music or a carer’s voice, delivered in direct response to the infant’s NNS [23]. Although both devices have demonstrated clinical relevance, their complexity, cost, and availability in certain regions may pose significant barriers to affordability and accessibility in hospitals and tertiary care units globally.

The developed device addresses the significant cost barrier of existing commercial systems, which price technologies like the Kangaroo™ NTrainer™ and Pacifier Activated Lullaby (P.A.L.) at over USD 4000 [24], making them inaccessible to neonatal and pediatric clinical units in low-income or developing countries. Unlike these solutions, the proposed system enables simultaneous and independent measurement of both expression (positive pressure generated by the infant’s lip force) and suction (negative intraoral pressure). Compared to NTrainer™ and P.A.L., the device offers superior portability through internal battery power and wireless Bluetooth data transmission, eliminating wired connections and facilitating use inside incubators. Its bottle-shaped design provides enhanced user-friendliness and reduces invasiveness in neonatal settings, while dedicated software specifically designed for healthcare personnel enables comprehensive data visualization and recording. Throughout its development, the device underwent rigorous analysis to meet electromedical safety requirements essential for clinical use.

Given the existing gaps in the objective assessment and comprehensive measurements of both stages of infants’ NNS within clinical settings, this study aims to present the design, development, and pilot phases of a device to objectively measure NNS skills. This paper describes the hardware and software aspects of the design and development phases. The correct operation of the device was clinically piloted with an initial data set collected from six newborns hospitalized within a neonatal intermediate care unit in a public hospital [Institutional Ethical Clearance CECSSVQ RES07/2017]. All caregivers provided written consent on behalf of their children to participate in the study [15].

## 2. Materials and Methods

### 2.1. Device for Measuring NNS

The device was developed using force-sensitive resistors (FSRs) to measure the expression stage and a differential pressure sensor to measure the sucking stage of NNS. Data were read by an analog-to-digital converter on a microcontroller and wirelessly transmitted via Bluetooth to a computer for graphical representation. Expression was measured using piezoresistive FSRs model 400 (Interlink Electronics, Irvine, CA, USA) [25]. These piezoresistive FSRs were placed inside a pacifier, where they came in contact with the infant’s oral cavity, capturing the force generated by the infant’s lips during the expression stage. Data were read by the analog–digital converter of a microcontroller and sent to a computer, where they were represented graphically for real-time interpretation. A MPX2050DP pressure sensor (NXP Semiconductors N.V., Eindhoven, NL) [26] was used to measure the sucking stage. This sensor measured the pressure changes generated between inputs and converted them into a voltage proportional to the pressure being applied. Lau and Kusnierczyk’s study [4] defined maximum and minimum levels of a newborn’s sucking pressure to be in the 0–160 mmHg range. The sensor used in this study had a pressure range between 0 and 375 mmHg (millimeters of mercury) [26]. The voltage delivered by the differential pressure sensor was conditioned by an AD623 amplifier (Analog Devices, Inc., Wilmington, MA, USA) [27]. This voltage was read by the microcontroller’s digital–analog converter and then sent to a computer for graphical representation.

**Figure 1:**
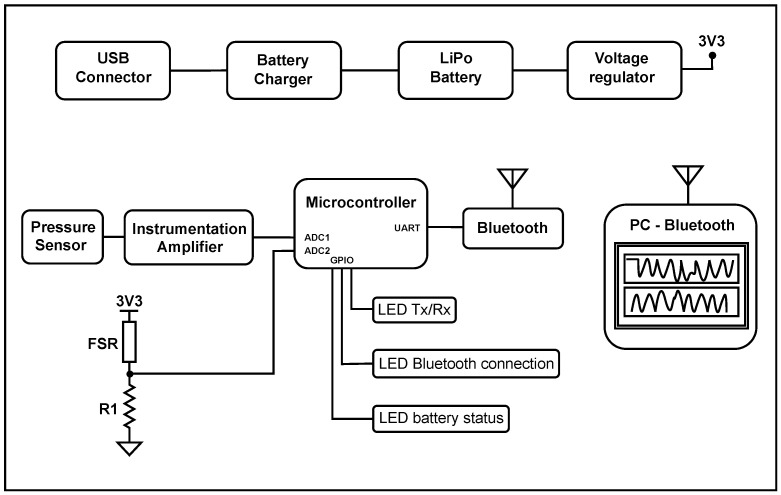
Sucking and expression measurement diagram. The voltage generated in the differential pressure sensor depends on the intensity of the sucking and is digitized and sent to the computer for graphic representation. The expression measurement consists of a voltage divider that changes the output voltage according to the force applied to the FSR sensors. The communication and transmission of data from the device to the computer were conducted through a wireless connection using a Bluetooth module. The upper section shows the power management and battery recharge system, which included a mini USB connector, an MCP73831 battery charger, a 3.7 V LiPo battery, and an LM3671 voltage regulator.

Figure 1 shows a detailed schematic of sucking and expression measurements. The technical specifications demonstrate robust data collection capabilities through a microcontroller’s analog-to-digital converter (ADC) [28] operating at a 20 Hz sampling frequency for both force-sensitive resistor sensors and differential pressure sensors, ensuring accurate capture of dynamic patterns characteristic of non-nutritive sucking in newborns. Wireless data transmission via an RN42 Bluetooth (Microchip Technology Inc., Chandler, AZ, USA) [29] module (power class 2) provides electrical isolation between the device and computer, enhancing safety through Universal Asynchronous Receiver–Transmitter communication protocols. The device’s power management and recharging system included a mini USB connector [30] for charging, an MCP73831 battery (Microchip Technology Inc., Chandler, AZ, USA) [31] charging module, a 3.7 V LiPo battery [32] as the power source, and an LM3671 voltage regulator (Texas Instruments Inc., Dallas, TX, USA) [33] to supply a stable 3.3 V to the circuit. These elements ensure continuous, safe operation of the system and enable battery recharging without removing the power source from the device. Additionally, the device included three indicator LEDs [34]: one signals correct communication between the device and the receiving laptop, another indicates the Bluetooth connection status (active or not), and a third LED displays the battery status or charge level.

### 2.2. Sucking Sensor Calibration

The MPX2050 has the characteristic of providing a linear voltage output, directly proportional to the applied pressure, and it is also “ratiometric to supply voltage.” This means that the output values delivered by the sensor for a given differential input pressure are proportional to the supply voltage. Considering this characteristic along with the linearity of its output, we concluded that the sensor’s sensitivity is also proportional to the supply voltage. This consideration was very important, as it allowed us to calculate the sensitivity value for any supply voltage within the range specified in the component’s datasheet.

It was important to ensure that the MPX2050 sensor provides output linearity with respect to both the supply voltage and the differential pressure applied to its inputs. To verify this hypothesis, measurements were conducted using the pressure sensor powered at three different supply voltages, and the corresponding output voltages were recorded. Various pressure values were applied using a syringe [35] and simultaneously measured by both the pressure sensor [26] and specialized pressure measurement equipment (Extech model PS115 (Extech Instruments, Nashua, NH, USA)) [36].

**Figure 2:**
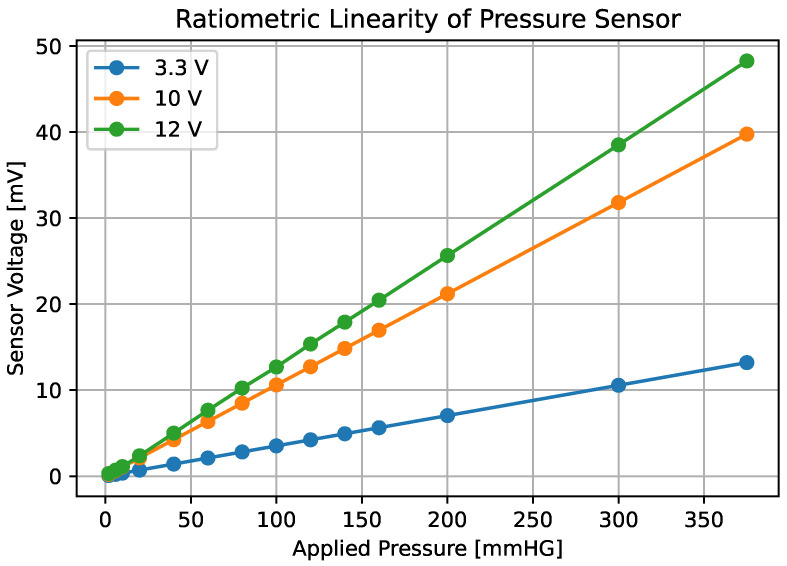
Graph of applied differential pressure versus output voltage. The green line corresponds to the sensor response when powered at 12 V, the red line when powered at 10 V, and, finally, the blue line when powered at 3.3 V.

Although the manufacturer specifies that the sensor is “ratiometric to supply voltage,” this condition was experimentally verified to guarantee a linear response in suction pressure measurements. To accomplish this, the first step was to supply the sensor with 10 V (nominal voltage indicated in its datasheet). Then, differential pressure applied at its inputs was progressively increased, and the output voltage was recorded at each increment. Subsequently, this procedure was repeated using supply voltages of 12 V and 3.3 V. The 12 V supply was arbitrarily selected to test linearity at voltages above the nominal rating, whereas 3.3 V was selected because it corresponds to the sensor’s supply voltage within the device. The results obtained are shown in Figure 2. It is important to note that pressure measurements below 160 mmHg were prioritized, as this was the range of interest for the low-cost device.

The NXP/MPX sensor [26] used to measure the sucking stage has a sensitivity proportional to the supply voltage, and this was determined as follows:

(1) $$\mathrm{Sensitivity}=\frac{3.3\cdot 0.1}{10}=0.033\left[\frac{\mathrm{mV}}{\mathrm{mmHg}}\right].$$

Considering that the maximum pressure to be measured in the sucking stage is approximately 160 mmHg [4] and the sensor’s sensitivity value from Equation (1), the approximate voltage value that the sensor would deliver for that pressure was calculated and is shown in the following equation:

(2) $$V=P\cdot 0.033=160\cdot 0.033=5.28\left[\mathrm{mV}\right].$$

Equation (2) determined that the voltage delivered by the pressure sensor when pressure was applied had a maximum of 5.28 [mV]. To amplify the voltage obtained in Equation (2), an instrumentation amplifier model AD623 was used. The maximum output voltage this amplifier can deliver is Vcc—0.5 V. Considering Vcc = 3.3 V, the maximum output voltage from AD623 is 2.7 V. The gain (G) was then determined and adjusted as follows:

(3) $$G=\frac{V_{\mathrm{out}}}{V_{\mathrm{in}}}=\frac{2.7\;V}{0.00528\;V}=511.$$

**Figure 3:**
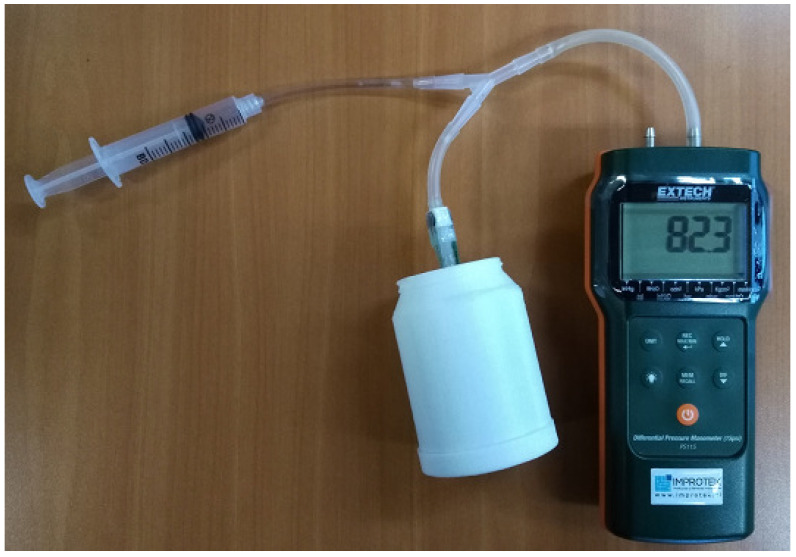
Connections of the equipment developed to an industrial calibrated digital manometer and syringe to generate negative pressure. The three components are joined by a plastic hose, generating a closed circuit without any loss of pressure.

The voltage obtained at the output of the instrumentation amplifier was read by the microcontroller’s digital-to-analog converter and sent to a computer, where it was converted to pressure and graphically represented. Once the transduction calculations for the differential pressure sensor were carried out, it was then calibrated by making the connection shown in Figure 3.

**Figure 4:**
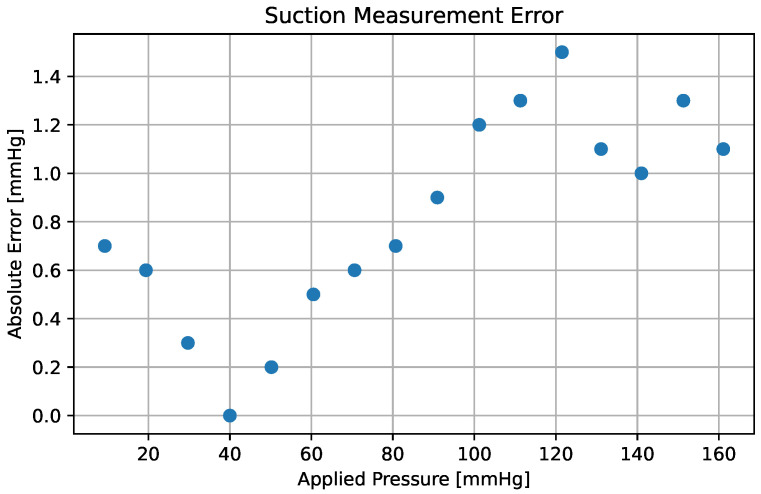
Measurement error (in mmHg) of the equipment developed with respect to the value delivered by the specialized equipment. The pressure was applied at 10 mmHg intervals.

Given that the hoses formed a closed circuit without leakage, the pressure throughout the circuit was identical. Therefore, when the syringe plunger moves backward, a negative pressure is generated and measured simultaneously by two pieces of equipment: the developed equipment and a specialized, calibrated one. To compare the developed equipment with the industrial calibrated digital manometer, 16 independent pressure value measurements were made and compared. Each independent pressure value was measured four times, and its average was calculated. The graphical output of the measurement error (in mmHg) is illustrated in Figure 4.

As observed in Figure 4, the maximum absolute error of measurement was 1.4 mmHg. Considering that the measurement value ranges between 0 and 160 mmHg, and the error corresponds to 1.2%, it was deemed that this variability/variance would not significantly affect the measurements performed.

### 2.3. FSR Calibration to Measurement of Expression

**Figure 5:**
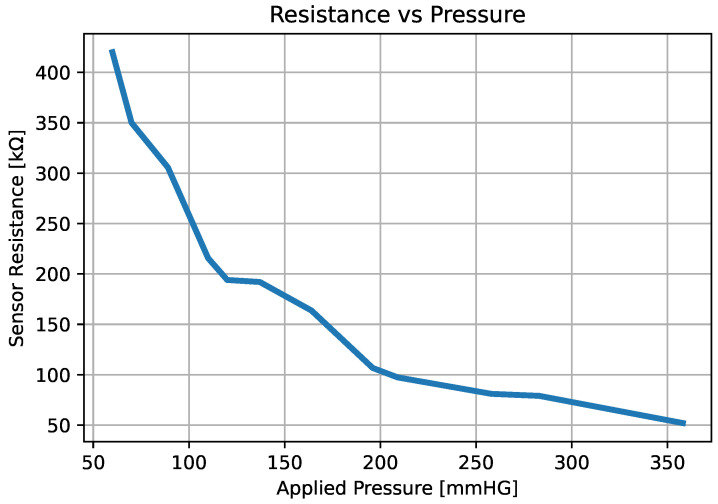
Force sensor response at different pressures.

**Figure 6:**
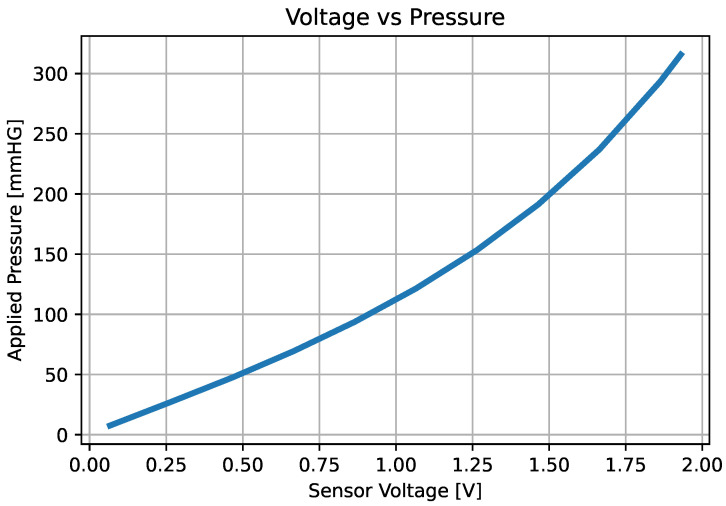
Pressure values obtained according to the voltage measured on the force sensor.

The resting electrical resistance of the force-sensitive resistor is greater than 10 MΩ when no force is applied, and its resistance decreases when a force is applied, reaching values close to 2 kΩ. To calibrate the force sensor, different masses were applied, and their resistance was compared to that of the sensor. For practical purposes, the applied weight was converted to pressure in mmHg. Figure 5 shows the response of the force sensor at different pressures. A potential trend line was adjusted to the values in Figure 6, and the value of the pressure applied to the sensor was determined by the resistance as follows:

(4) $$P=6\times 10^{6}\times R_{FSR}^{-0.882}.$$

RFSR is the resistance that the sensor expresses in Ohm and P is the pressure measured in millimeters of mercury. To measure the resistance of the force sensor, a voltage divider was used. The voltage was measured to estimate the force intensity applied to the sensor. For R1, a resistance of 100 KΩ was used, as it was closer in value to the one the FSR delivers at half the measured scale. The output voltage was defined as follows:

(5) $$Vo=\frac{R1}{R_{fsr}+R1}\cdot V.$$

A 100 KΩ resistor was used, and the circuit was powered by 3.3 V. Then, the $R_{fsr}$ value was defined according to

(6) $$R_{fsr}=\frac{1\times 10^{5}\times 3.3}{V_{o}}-100,000.$$

In summary, we have a function that determines the resistance of the sensor as a function of the voltage generated at its ends. Figure 6 shows a summary of the pressure values obtained from the application of Equation (6), which corresponds to each output voltage value obtained as a function of the resistance of the FSR.

### 2.4. Design and Printed Circuit Board Development and Sensor Integration

**Figure 7:**
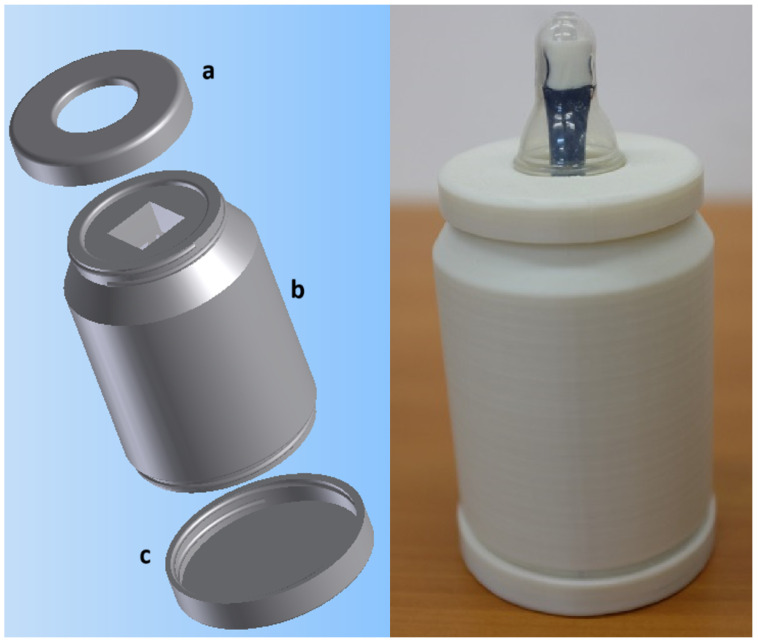
Designed prototype parts: (a) front cover, (b) main body, and (c) rear cover on the **left**. Prototype device ready to use on the **right**.

The developed prototype device resembles a traditional feeding bottle and consists of three pieces: the front cover, main body, and rear cover. These three pieces are shown in Figure 7 on the left.

**Figure 8:**
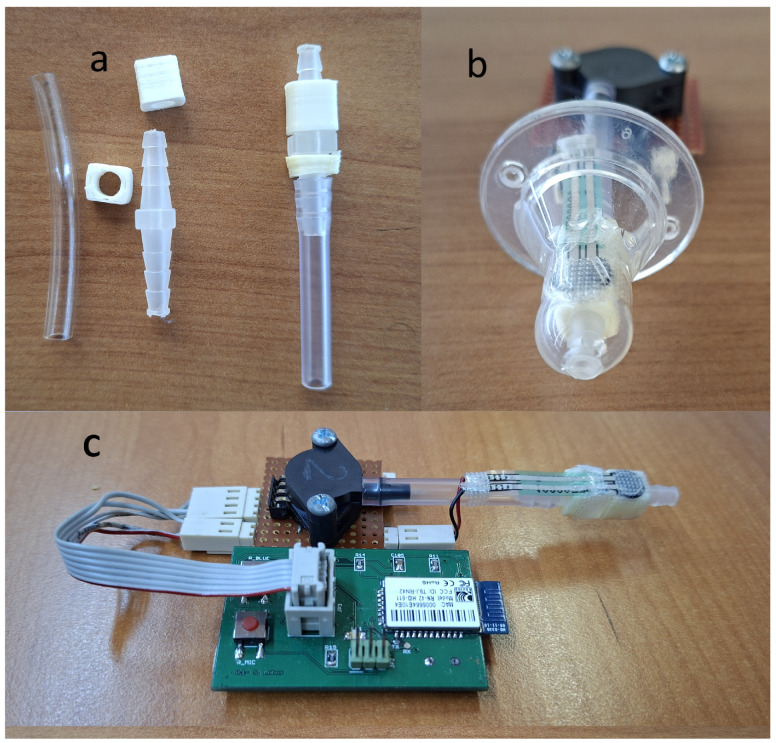
Internal components and assembly of the sensing device designed for intraoral pressure and force measurements. (**a**) Exploded view of the rigid conduit (internal diameter: 3 mm), connector elements, and custom-designed mounting piece. The rigid conduit maintains a direct connection to the atmosphere or to the infant’s oral cavity without deformation under external forces, ensuring unobstructed airflow and accurate intraoral pressure measurement. (**b**) Frontal view of the device assembled with the pacifier, illustrating the integration of the pressure sensor and FSR sensors and the connection of the teat (pacifier) to the device. A strategically positioned hole at the top of the pacifier enables a direct connection between the rigid conduit and the internal pressure sensor, ensuring precise transmission of intraoral pressure from the infant’s oral cavity. (**c**) Top view of the assembled system, showing the arrangement of the custom mounting component, which provides a flat surface for the FSR sensors and incorporates a mechanism that ensures a unique orientation and prevents assembly errors after sterilization. This architecture enables both physical and electrical isolation between the pressure sensor and the FSR sensors, allowing for independent and interference-free measurements from both sensor types while maintaining a reliable connection to the infant’s oral cavity. The cable is visible, featuring a polarized header connector for connection to the PCB. This connector mates with the vertical header on the PCB shown on the left in Figure 9.

**Figure 9:**
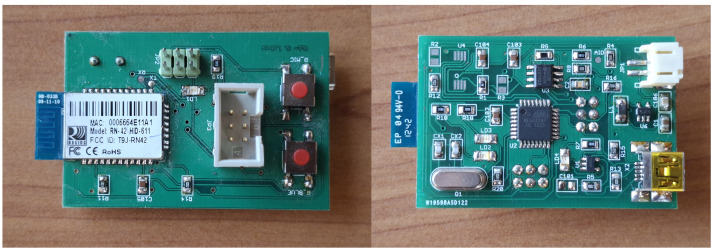
Manufactured electronic board. The PCB dimension is 5 by 3.5 cm.

The device architecture incorporates design elements to ensure measurement accuracy and clinical safety. Physical and electrical isolation between the pressure sensor and force-sensitive resistor sensors prevents interference, while a rigid conduit with a 3 mm internal diameter directly connects the pressure sensor to the infant’s oral cavity (Figure 8a). This non-deformable conduit resists external forces and maintains unobstructed airflow, eliminating measurement artifacts and ensuring accurate intraoral pressure detection. A component specifically designed to provide a flat mounting surface for the FSR force sensors was positioned on top of this rigid connector (Figure 8c). This component features a unique locking mechanism that allows it to be placed in only one orientation, thereby preventing assembly errors after sterilization procedures, ensuring consistent sensor placement, and enabling independent, non-interfering measurements from both the FSR sensors and the pressure sensor, as well as from the pressure sensor’s connection to the infant’s oral cavity. All pacifiers used have an opening at the top (Figure 8b), which enables the rigid cylinder to connect directly to the infant’s oral cavity, ensuring that intraoral pressure is faithfully transmitted to the pressure sensor. The force exerted by the infant’s lips on the FSRs does not deform or interfere with the rigid conduit, thereby enabling simultaneous and independent measurement of intraoral pressure and expression force. Critical safety considerations shaped every design element, particularly for components in direct contact with the infant’s oral cavity that require saliva contamination resistance and sterilization capability. This design priority prevents cross-contamination between infants in neonatal and pediatric care units. Circuit board connectors underwent careful selection to eliminate reversible connections while maintaining safety during airflow conduction and sterilization procedures. The electronic board developed and assembled is shown in Figure 9, and the fully assembled prototype is shown in Figure 7.

The assembled device weighs 260 g with a volume equivalent to a standard 200 mL baby bottle, enabling one-handed operation by clinical staff, who can hold it in one hand during measurement and easily place it inside an incubator. This design facilitates use with premature infants and in hospital environments, allowing for practical and safe measurement of sucking behavior without causing additional discomfort or interference for the newborn.

### 2.5. Asepsis

During the development of the device, a critical element in ensuring the safe usage of the device was considered: avoiding cross-contamination between infant users. As such, the components of the equipment that are in contact with the saliva/fluids of an evaluated infant are removable, which allows these components to be sterilized. The differential pressure sensor was sterilized using a hospital-grade autoclave. The manufacturer recommends a maximum operating temperature of 125 °C for the MPX2050 sensor, while sterilization is performed at 120 °C. Therefore, the pressure sensor can withstand sterilization. Given that plastic connectors do not tolerate high temperatures, they were sterilized with ethylene oxide [37]. The maximum temperature reached by this type of sterilization is approximately 60 °C and is unlikely to damage the plastic hoses. Force-sensitive resistors were also sterilized with ethylene oxide, as the manufacturer indicates a maximum working temperature of 80 °C. The equipment housing was cleaned between each infant with an ammonia-based hospital-grade surface cleaner [38], a disinfectant widely used within hospital settings. Finally, pacifiers [39] were disposable and changed between each infant.

### 2.6. Software

**Figure 10:**
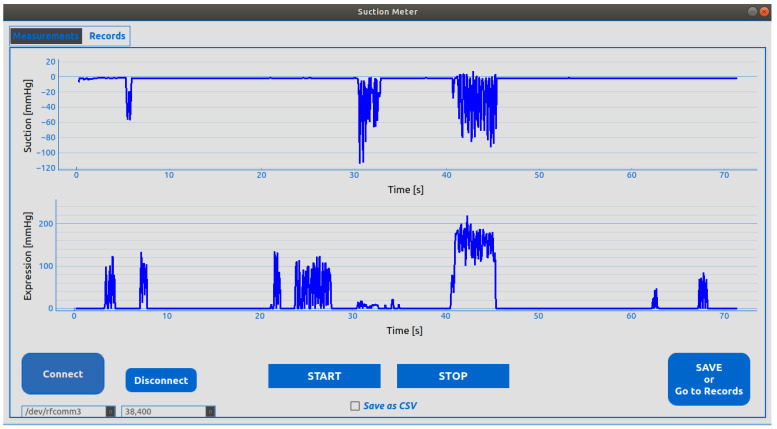
Graphic interface screen displaying the data received from the device. Start, stop, and save buttons are displayed at the bottom of the screen.

**Figure 11:**
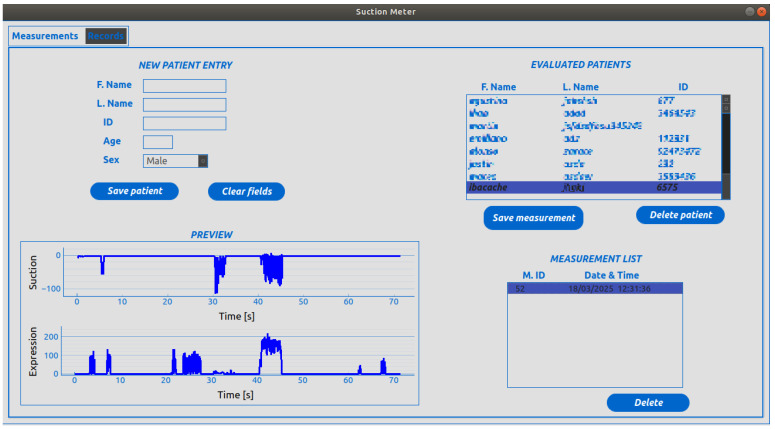
Patient record screen that allows clinicians/researchers to enter and save patients’ data and display infants’ NNS measurements.

To record the data obtained during the measurement of sucking and expression stages, a graphic interface was designed to receive data from the device via Bluetooth and graph the data in real time. This interface had two windows where the curves obtained in the measurement of sucking and expression stages were generated. In the top window, the graph corresponding to the sucking measurement was shown, while the graph representing the expression measurements was displayed at the bottom of the graph (see Figure 10). Additionally, an SQLite database version 3.40.1 was implemented to facilitate the storage and management of patient demographics and NNS measurements for each infant. The screen where information was stored in the database is displayed in Figure 11.

### 2.7. Availability and Cost

For the development of this low-cost NNS measuring device, 50 components were used, with prices ranging from USD 0.10 to USD 19. The most expensive components include the following: 8-bit AVR microcontroller from Atmel (ATMEGA328P-AU, USD 2.97), low-power instrumentation amplifier (AD623ARZ, USD 7.63), high-precision battery monitor with I2C interface (Maxim Integrated) (DS2782E+, USD 9.71), Class 2 Bluetooth Module (Microchip) (RN42U-I/RM, USD 18.04), differential pressure sensor (NXP/Freescale) (MPX2050DP, USD 17.12), piezoelectric or tactile sensor (30-49649, USD 5.86), and LiPo battery module with JST connector (SparkFun) (PRT-13854, USD 13.61). The total material cost was USD 91.03.

To this value, PCB manufacturing costs (USD 22.00) and 3D printing materials (USD 3.46) were added, resulting in a total estimated cost of USD 117 per unit. This represents low production costs, although packaging, certification, and logistics expenses are not included. Costs could be further reduced through economies of scale resulting from mass production. The device’s affordability becomes particularly evident when compared to commercial models currently available, which are priced above USD4,000 [24], representing a cost difference of approximately 34 times the price. The developed device offers a commercially competitive alternative that significantly increases accessibility for resource-limited hospitals and neonatal care units, particularly in low- and middle-income countries.

## 3. Results

Pilot data were obtained from six premature newborns hospitalized in a neonatal care unit in a public hospital. The assessment consisted of placing the device inside the infant’s oral cavity and measuring their NNS skills. Measurements were recorded on the computer through the device software and included newborns’ sucking and expression stages. All assessments were conducted at a time close to the infants’ feeding schedule and after their routine checkup, which allowed infants to be awake and ready to be fed (e.g., hungry and alert).

**Figure 12:**
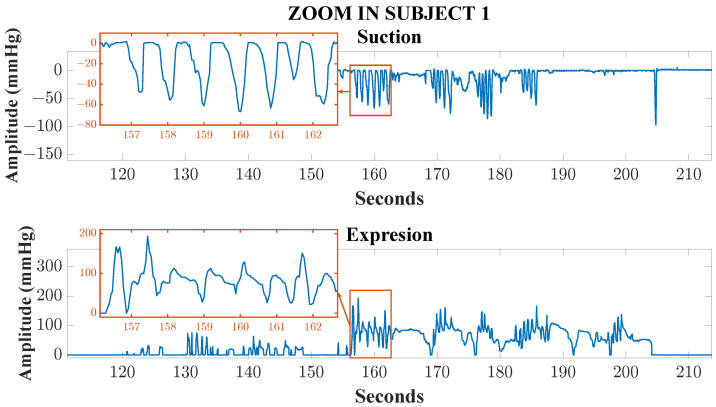
Expanded segment of simultaneous recordings of suction (**top**) and expression (**bottom**) in a hospitalized preterm newborn, acquired with the developed device. The characteristic morphology, including distinct cycles, transients, peak amplitudes, frequency, and pauses between events, can be clearly observed. This level of signal fidelity enables accurate clinical classification of non-nutritive sucking skills according to the Lau and Kusnierczyk [4] scale.

To demonstrate the fidelity of the developed system, Figure 12 shows a short time segment of suction and expression recordings from a preterm newborn. This waveform demonstrates the device’s ability to clearly capture key signal features with the characteristic morphology of individual cycles of non-nutritive sucking and expression, allowing for the distinction of transients, peak amplitude values, frequency, and pauses between events. This level of fidelity is sufficient for the clinical classification of non-nutritive sucking skills according to the scale proposed by Lau and Kusnierczyk [4], since this classification method is based on the analysis of global patterns, such as the coordination between phases and the frequency of bursts, all of which are adequately represented with the sampling rate used in this system.

**Figure 13:**
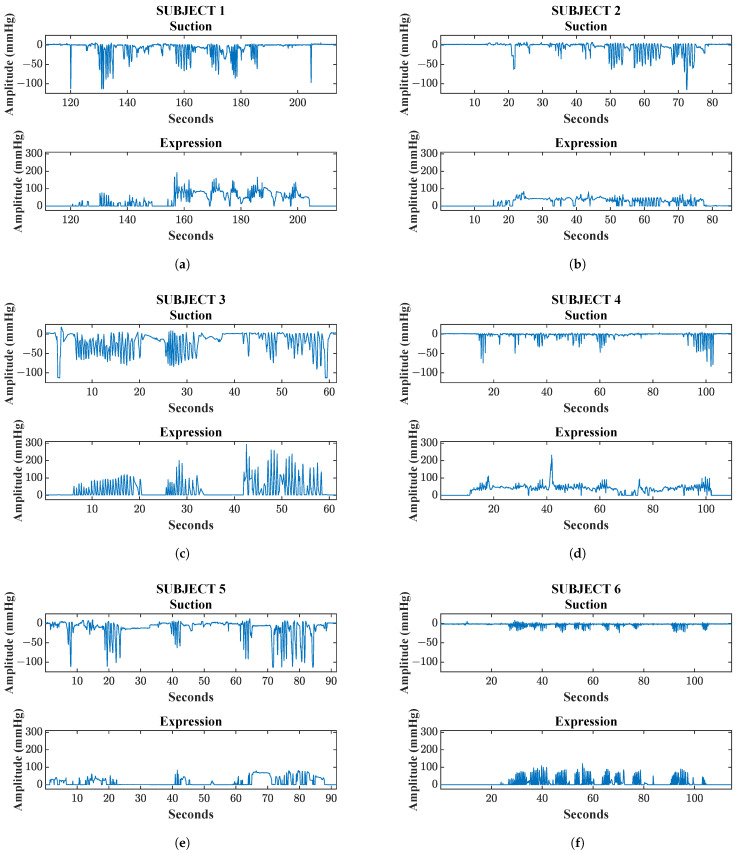
Pilot data obtained from six premature newborns hospitalized in a neonatal care unit, illustrating suction and expression stage patterns measured by the developed device. Each plot (**a**–**f**) corresponds to an individual subject’s recorded data. Suction plots show negative amplitude values (mmHg), indicating the intensity of sucking bursts, while expression plots show positive amplitudes (mmHg), indicating compression movements.

Data analysis focuses on three main measurement aspects: first, quantification of sucking bursts (defined as continuous sequences of sucks without pauses [40]) and sucks per burst; second, assessment of maximum intensity for both sucking and expression stages; and third, evaluation of coordination patterns between the two phases: synchronization, rhythmicity, and alternation between sucking and expression occurred. These analyses formed the basis of the classification system developed by Lau and Kusnierczyk [4] to discriminate between the five stages of NNS development. All the infants assessed showed weak NNS: no infant reached a sucking intensity of 160 mmHg, which is the maximum value for established sucking (at approximately the term age). Example plots depicting measurements obtained concurrently from individual subjects are shown in Figure 13, and summary statistics of relevant parameters are displayed in Table 1.

In the sucking plot for Subject 1, six bursts were observed with a duration of approximately 6 s each, with 5–7 sucks per burst, and a maximum intensity of −120 mmHg. In the expression plot, seven bursts of 4–14 s were observed, with a maximum intensity of 180 mmHg, consistent with Stage 4 of Lau and Kusnierczyk’s [4] classification system.

In Subject 2, five bursts of 3–9 sucks per burst were observed, reaching a maximum sucking intensity of −120 mmHg. The expression plot shows five bursts and a maximum value of 90 mmHg. During the first two bursts, this newborn did not coordinate the suction and expression stages, showing limited coordination between the two stages. In the last three bursts, a coordinated and rhythmic movement was seen, consistent with Stage 4.

In Subject 3, there were three bursts with 14–23 sucks per burst. The maximum intensity of the sucking stage was observed twice, occurring at 2 and 59 s in the assessment (−110 mmHg). The expression plot exhibits three bursts and a maximum value of 280 mmHg. This measurement is classified as Stage 5, since sucking and expression were well-formed, and coordinated and rhythmic movements between sucking and expression were observed in all bursts across the two stages.

Plots for Subject 4 were classified as Stage 3B, as the maximum intensity for sucking and expression stages were −75 mmHg and 200 mmHg, respectively. The characteristics of the expression stage were maintained over the measurement period, indicating that rhythmicity and alternation between the sucking and expression stages were not yet fully developed. Four bursts were observed in plots for Subject 5. The maximum intensity of −115 mmHg was reached in the second and last burst for the sucking stage, and 80 mmHg was the maximum reached for the expression stage. Expression and sucking stages were occasionally coordinated. However, there were bursts where expression remained steady but sucking was inconsistent. The classification of this infant matched Stage 3B in Lau and Kusnierczyk’s [4] classification system.

Lastly, in Subject 6, eight sucking bursts of different durations were observed, with similar intensities of approximately −20 mmHg. Given that the maximum intensity was low, this infant’s NNS capacity may have not been well developed. As for the expression stage, it was more frequently observed than sucking. According to Lau and Kusnierczyk’s [4] classification system, this would be a distinctive characteristic in the NNS of infants with immature feeding skills. Table 1 shows a summary of the most relevant parameters for each of the six assessed infants.

## 4. Discussion

During the development of this device, design considerations were incorporated to allow the internal sensors and plastic connectors to be easily replaced between each use and sent for sterilization. This consideration presented a significant challenge during the design and development phases.

During preliminary testing, it was found that infants who were being breastfed expressed some dislike for using the device and some rejected it. This dislike may have been due to the differences between the breastfeeding and bottle-feeding experiences, including underlying biological and psychological mechanisms associated with breastfeeding that are beyond the scope of this study.

The device supported health professionals in decision-making using quantitative data about infants’ NNS parameters. The use and handling of the device did not present additional challenges to the professional assessing the newborns. The device was capable of holding both sensors (i.e., sucking and expression) and presented no issues when replacing the pacifier between subjects or in the removal of sensors for sterilization. Further, sterilization did not damage sensors or any internal components. This was corroborated by comparing the electrical parameters before and after sterilization. Regarding the measurement of the sucking stage, the sensors exhibited an adequate response to pressure variations, indicating the clinical suitability of the device. Similarly, the force-sensitive resistors were found appropriate and responsive in the measurement of the expression stage of NNS skills.

Given that the six infants assessed in the present study were born prematurely and admitted to an intermediate neonatal care unit, their assessments were mostly characterized by immature and disorganized NNS sucking skills. Immature NNS was also reported in Lau and Kusnierczyk’s [4] study and included frequent alternation between stages, with increased rates and pace observed in both sucking and expression. All six subjects in this study were born prematurely, and their NNS skills showed immature patterns, as measured by this device. Objective assessment of NNS is essential for determining feeding readiness in infants admitted to neonatal and pediatric care units. This information supports informed clinical decision-making about each infant’s oral-motor development and guides evidence-based management and intervention, especially in a population that continues to undergo neurological development outside the womb until reaching full-term age. Future work should, therefore, compare the results between premature newborns and full-term newborns to determine differences in pressure generated within the oral cavity and further characterize premature newborns’ NNS, as well as the NNS in term newborns who exhibit some feeding difficulty. Lastly, future research projects may explore the objective assessment of nutritive sucking in infants to support clinical decision-making and management of both inpatient and outpatient children who experience feeding difficulties.

Clinical validation through a pilot observational study demonstrated the device’s ability to accurately and reproducibly measure non-nutritive sucking in preterm neonates under real hospital conditions. The main objective was to evaluate the technical and clinical performance of the system rather than to perform direct clinical comparisons between preterm and term neonates. Future studies are planned to include larger sample sizes of preterm neonates at different gestational ages and broader clinical applications to assess the generalizability of findings. These future studies will also include an in-depth exploration of the effects of the sampling frequency to determine if it is necessary to increase this frequency to detect signal features that are currently not described in the literature.

## 5. Conclusions

We developed a simple, cost-efficient, and versatile medical device and software that clinically and safely measure infants’ NNS skills in real time. This device could be used for the objective quantification of NNS parameters within clinical settings and be used as an aid to support clinical decision-making, such as determining readiness for feeding among premature and full-term newborns and infants hospitalized in neonatal units, discerning the need for oro-motor intervention by speech–language therapists, and measuring outcomes relevant to the management of newborns and infants with feeding difficulties.

The results obtained in this study are similar to those reported by Lau and Kusnierczyk [4], who illustrated that NNS progressively develops through changes in the patterns and intensity of an infant’s sucking and expression stages. Therefore, the developed device is effective in supporting the quantitative and objective assessment of an infant’s NNS and can be used by specialized professionals to inform clinical decision-making within neonatal and/or pediatric units. This device and software may also be used for training students and professionals from relevant health disciplines and potentially support the education and coaching of caregivers of infants with feeding difficulties.

This study showed that the pressure sensors used are suitable for the measurement of a newborn’s sucking and expression stages and are capable of responding to and discriminating between pressure changes and variations. Similarly, the force-sensitive resistors used to measure the expression stage were able to determine changes in the pressure generated by newborns. The device developed held both sensors and presented no problems when parts such as the pacifier were replaced or sensors were removed for sterilization. Additionally, sterilization processes did not damage the sensors or internal components of the equipment, which is crucial in the design and development of biomedical devices.

Regarding the assessments conducted, all six infants presented with immature and disorganized NNS skills, demonstrating an increased alternation between sucking and resting. Likewise, the sucking stage lasted longer than one suction per second, which has been described as a mature pattern. These characteristics have been previously described in the literature in newborns with immature sucking skills.

Future research should measure and compare NNS between premature and term newborns to determine the impact of prematurity and other health conditions on children’s feeding skills. In addition, future studies could investigate the automation of the device in such a way that NNS assessment and measurement within clinical units are not reliant on a clinician’s experience with subjective NNS assessment before the use of a device like this.

## 6. Patents

The design of this device for measuring non-nutritive sucking in newborns is protected by the patent number CL2016003353 [41].

## Figures and Tables

**Table 1:** Summary of the most relevant parameters of each measurement.

Subject	Number of Sucking Bursts	Number of Expression Bursts	Maximum Sucking Intensity (mmHg)	Maximum Expression Intensity (mmHg)
Subject 1	6	7	−120	180
Subject 2	5	5	−120	90
Subject 3	3	3	−110	280
Subject 4	5	2	−85	220
Subject 5	4	4	−115	80
Subject 6	8	8	−20	100

## Data Availability

The data are contained within the article.
